# Identification of phytochemical as a dual inhibitor of PI3K and mTOR: a structure-based computational approach

**DOI:** 10.1007/s11030-022-10541-2

**Published:** 2022-10-16

**Authors:** B. Harish Kumar, Suman Manandhar, Sneha Sunil Choudhary, Keerthi Priya, Tanvi V. Gujaran, Chetan Hasmukh Mehta, Usha Yogendra Nayak, K. Sreedhara Ranganath Pai

**Affiliations:** 1 Department of Pharmacology, Manipal College of Pharmaceutical Sciences, Manipal Academy of Higher Education, Manipal, 576104 Karnataka India; 2 Department of Pharmaceutics, Manipal College of Pharmaceutical Sciences, Manipal Academy of Higher Education, Manipal, 576104 Karnataka India

**Keywords:** PI3K/mTOR pathway, Molecular dynamics, Structure-based computational approach, Salvianolic acid A

## Abstract

Breast cancer is a common form of cancer that affects both men and women. One of the most common types of genomic flaws in cancer is the aberrations in the PI3K/AKT/mTOR pathway. The benefit of dual targeting PI3K as well as mTOR is that the kinase-positive feedback loops are more effectively inhibited. Therefore, in the current study, structure-based models like molecular docking, MM-GBSA, Qikprop, induced fit docking, simulated molecular dynamics (MD), and thermal MM-GBSA were used to identify the phytochemicals from the zinc 15 database, which may inhibit PI3K and mTOR. After docking the phytochemicals with PI3K (PDB 4FA6), ten ligands based on the docking score were selected, among which salvianolic acid C had the highest docking score. Hence, salvianolic acid A was also docked. All the ligands taken showed a binding energy of greater than − 30 kcal/mol. The predicted ADME showed that the ligands have druggable properties. By performing MD of the top five ligands and salvianolic acid A, it was found that ZINC000059728582, ZINC000257545754, ZINC000253532301, and salvianolic acid A form a stable complex with PI3K protein, among which ZINC000014690026 showed interaction with Val 882 for more than 89% of the time. Salvianolic acid A is already proven to suppress tumor growth in acute myeloid leukemia by inhibiting PI3K/AKT pathway, but the exact protein target is unknown. Therefore, the present study identifies new molecules and provides evidence for salvianolic acid A for dual inhibition. Further experiments must be performed both in vitro and in vivo to support the predictions of these computational tools.

**Graphical abstract:**

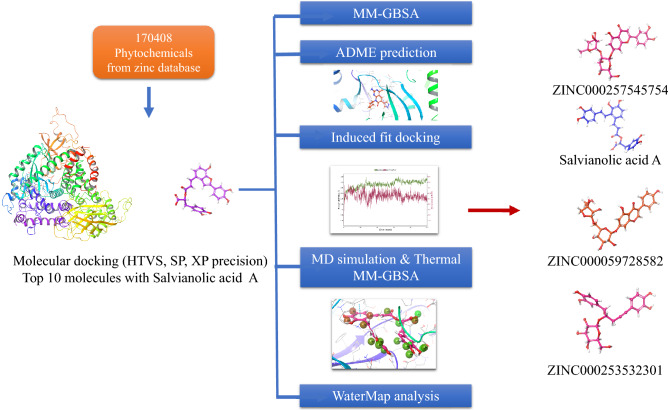

## Introduction

Breast cancer affects both men and women, with a peak incidence varying between people of different regions. The incidence of breast cancer in Asia is between the age of 40 and 60 years, and in western countries it is between 60 and 70 years [1]. It is a leading cause of cancer deaths in this demographic globally. Albeit uncommon, breast cancer can affect even men. About 1 in 100 men (< 1%) is diagnosed with breast cancer [2, 3]. The crucial risk factors are family history, age, early or late menopause, and nulliparity. Other lifestyle factors like alcohol consumption, lack of physical activity, overweight, etc., also play a pivotal role in the occurrence of cancer [3, 4]. Based on the hormonal receptors, breast cancer can be categorized as a luminal subtype [estrogen receptor-positive (ER+) or progesterone receptor-positive (PR+)], a Her2-enriched subtype (Her2+; overexpressed Her2 receptor), and triple-negative (TNBC)/basal-like subtype (ER−, PR−, and Her2).

The pathway that is frequently activated in most cancer cases is the PI3K/Akt/mTOR pathway. It regulates cell survival, cell division, migration, cellular protein synthesis, glucose metabolism, and immune regulation [5]. Studies have shown that two crucial genes of this pathway, PI3K catalytic subunit α (PIK3CA) and phosphatase and tensin homolog PTEN are the most frequently altered ones in breast cancer. Oncogenic activation in TNBC can be due to upregulation of upstream regulators like epidermal growth factor receptor (EGFR), underexpression of proline-rich inositol polyphosphatase, dysfunction or underexpression of PTEN, and activation of PI3K catalytic subunit α (PIK3CA). As a result, PI3K can also be a potential target in treating TNBC. PI3Ks have an important role in the cascade that leads to tumor cell growth. PI3Ks contain catalytic (p110) and regulatory (p85) subunits that can exist in four isoforms (α, β, δ, and γ) [6]. They are classified into Class I, II, and III based on their specific different activation and downstream molecules. PTEN is a cellular antagonist for the PI3K activity with a lipid phosphatase activity, and it reduces the amount of PIP3 in the cell, hence playing the role of tumor suppressor [7].

Across different breast cancer subtypes, the most common abnormalities are aberrations in the PI3K/AKT/mTOR pathway. Therefore, in these tumors, greater than 70% harbor gene amplification or mutations in the pathway. Stimulation of this pathway is by the ligands or growth factors that are specific to receptor tyrosine kinases (RTKs) that also include EGFR, insulin, insulin-like growth factor 1 (IGF-1) receptor, fibroblastic growth factor, etc. [8].

Downstream to this pathway is mTOR, which is the complex that sets different biological functions in motion without a doubt, and is a well-studied target^.^ [9, 10]. mTOR is a Ser/Thr kinase and a part of PI3K superfamily called class IV PI3K. It is present as two complexes, mTORC1 and mTORC2. mTORC1 constitutes the catalytic subunit, a regulatory associated protein of mTOR (Raptor), proline-rich AKT substrate of 40 kDa (PRAS40), and mLST8/GbL protein. mTORC2 constitutes rapamycin-insensitive of mTOR (Rictor), mammalian stress-activated protein kinase interacting protein 1 (mSIN1), and mLST8/GbL. There are well-established drugs such as rapamycin for inhibition of mTORC1. But still, mTOR is also a potential target because of the mTORC2 complex, which acts as a PDK2 that phosphorylates AKT which is required for a complete activation at Ser473 at carboxyl terminal. However, the role of PDK in tumor progression is unknown; this activation is required for tumor growth [11]. Thus, an mTOR inhibitor that targets mTORC1 and mTORC2 would inhibit PI3K pathway activation more efficiently than rapamycin [10, 12].

Various inhibitors of pan-PIK3 and mTOR pathways are individually approved in cancer treatment, and clinical trials for others are underway. Some of the approved inhibitors of PI3K are Idelalisib, Copanlisib, and Duvelisib. Approved mTOR3 inhibitors are rapamycin and its analogs (rapalogs) like everolimus, sirolimus, and temsirolimus as first-generation inhibitors. Currently, second-generation inhibitors are in clinical trials. However, a major setback of these inhibitors is the poor response clinically, and resistance to the pathway inhibition [8].

Targeting PI3K and mTOR simultaneously inhibits the receptor tyrosine kinase-positive feedback loops more efficiently than PI3K alone. Thus, the dual inhibitors of PI3K and mTOR can simultaneously inhibit the pathway both upstream and downstream [7]. Another advantage of using a dual inhibitor is the lower chance of resistance generation [11, 13]. Researchers like Alejandra ortiz gonzále et al. [14], by using in silico tools, predicted the effect of Opuntia joconostle in various models of breast cancer for its antiproliferative property through PI3K/AKT/mTOR pathway, proving that computer-based prediction can be beneficial for identifying lead molecules for cancer. Therefore, the current study tries to identify a phytochemical with dual specificity for both PI3K and mTOR, which can be an effective molecule in the treatment of cancer.

## Methodology

The computational studies were performed on a Linux-based system with 8 GB RAM using Maestro Schrödinger suite version 2018–3 (Schrödinger, LLC, New York). Tools like, Ligprep, Protein Preparation Wizard, GLIDE, Desmond, and WaterMap were used for the study.

### Protein selection and preparation

Based on the literature, PI3K PDB 4FA6 has inbound PI3K, and mTOR dual inhibitor, 2-amino-8-cyclopentyl-4-methyl-6-(1H-pyrazol-4-yl)pyrido[2,3-d]pyrimidin-7(8H)-one, and extensive computational work has been done. Therefore, the same PDB protein was used in this study. The 4FA6 PDB was retrieved from the RCSB database and processed with Protein Preparation Wizard [15]. The missing amino acid residues, hydrogen bonds, and side chains were added during protein preparation. The ionization state of the het group at pH 7.4 ± 0.5 was generated, H-bonds were assigned and optimized, and water molecules that are not essential for ligand binding (beyond 3.0 Å from het groups) were removed. In the end, the protein structure was minimized to the lowest energy level by using the Optimized Potentials for Liquid Simulations (OPLS3e) force field.

### Ligand preparation

In this study, natural product molecules (170,408) obtained after applying a filter in the availability category “now” from the Zinc 15 database [16] were downloaded. After importing the molecules, they were optimized using the Maestro suite Ligprep tool to obtain appropriate three-dimensional structures optimized with the ionization state at pH 7.4 ± 0.5. The three-dimensional structures were used to determine the chirality of the molecules, and minimization was done using Optimized Potentials for Liquid Simulations force field.

### Molecular docking and MM-GBSA calculation

Molecular docking was performed by using Glide module [17]. First, a grid of size 20 Å was generated centroid to the inbound molecule 2-amino-8-cyclopentyl-4-methyl-6-(1H-pyrazol-4-yl)pyrido[2,3-d]pyrimidin-7(8H)-one. The receptor grid file was loaded, and the inbound ligand was redocked into the generated grid to validate the grid by calculating the root mean square deviation (RMSD) of pose generated before and after the docking. After validation, the prepared ligands of the zinc 15 database were selected from the project table to dock with the protein. Initial docking was performed in high-throughput virtual screening (HTVS) mode (123,940 molecules), standard precision (5214 molecules), and extra precision (153 molecules). The ligand poses after docking were then analyzed for interaction patterns with the protein. The ligand poses showing the required interactions with the protein were selected. The top ten hits obtained were analyzed for ligand pose and interaction with protein. After that, further evaluation was done by using molecular mechanics generalized Born surface area (MM-GBSA).

The prime MM-GBSA [18] uses the VSGB solvent model and OPLS3e force field. More negative Kcal/mol values indicate stronger binding, as MM-GBSA binding energies are approximate binding free energies.

### Predicted pharmacokinetic property

The ADME properties of the selected phytochemicals were predicted by using the QikProp module. Drug-likeness of phytoconstituents was evaluated by using various descriptors and pharmacokinetic properties like predicted octanol/water partition coefficient (QPlogPo/w), predicted aqueous solubility (QPlogPo/w), predicted apparent Caco-2 permeability (QPPCaco), predicted brain/blood partition coefficient (QPlogBB), Lipinski’s rule of 5, and human oral absorption.

### Induced fit docking

Induced fit docking (IFD) [19] was performed using Maestro for the selected top five ligands obtained from molecular docking and MM-GBSA. In docking studies, the amino acid interacting with ligands is rigid, which is not the case in physiological conditions. Therefore, IFD is used to permit flexibility in the protein. During IFD, the ligand and receptor van der Waals scaling was maintained at 0.50. Calculations were performed using the standard precision protocol, generating a maximum of twenty poses of the protein with ligands. All poses were analyzed individually, and the pose in which the maximum number of interactions was seen with key residues was taken forward for molecular dynamic simulation.

### WaterMap analysis

To have a better understanding of water molecules’ role present within the binding pocket of 4FA6, which may affect binding of ligands, the WaterMap module [20] from Maestro suite was used. The inbound ligand in the protein was defined as the region of interest for analysis, and the water molecules within 5 A° were analyzed. Before running the simulation for two nanoseconds using the OPLS3e force field, all the water molecules within the crystal structure were eliminated. After simulation, the selected ligands were evaluated for the possible overlapping of the hydration site and Gibbs energy of the water molecule.

### Molecular dynamics simulation and thermal MM-GBSA

MD simulation was performed for the top five shortlisted after extra precision docking and MM-GBSA results. The Desmond module [21] of Schrödinger was used initially. Periodic boundary condition (PBC) of size of 10 × 10 × 10 Å with orthorhombic shape was prepared around the protein complex and solvated using simple point charge (SPC) as a solvent model. At this stage, the iso-osmotic condition was maintained by adding sodium and chloride ions. The solvated system was minimized using the 2000 maximum iterations and 1.0 (kcal/mol/Å) convergence threshold for 100 ps. Finally, the solvated system was used in MD simulation at NPT (Normal pressure and temperature) ensemble for 100 ns at 1.01 bar pressure and 300 K temperature. A simulation interaction diagram was generated to analyze the results.

To understand the binding energy of the ligands in different frames, MD simulation and MM-GBSA were carried out, in which every tenth frame of 1002 frames generated from MD simulation was used for calculating binding energy. The binding energies of the ligands were calculated by the formula mentioned below.

$$ {\text{MMGBSA }}\;{\text{dG}}\;{\text{ Bind }}\;\left( {{\text{NS}}} \right) \, = {\text{ Complex }}\;{-}{\text{ Receptor}}\; \, \left( {{\text{from }}\;{\text{optimized }}\;{\text{complex}}} \right) \, {-}{\text{ Ligand}}\; \, \left( {{\text{from }}\;{\text{optimized }}\;{\text{complex}}} \right) $$

## Results and discussion

### Molecular docking and selection of ligand based on binding affinity

**Fig. 1:**
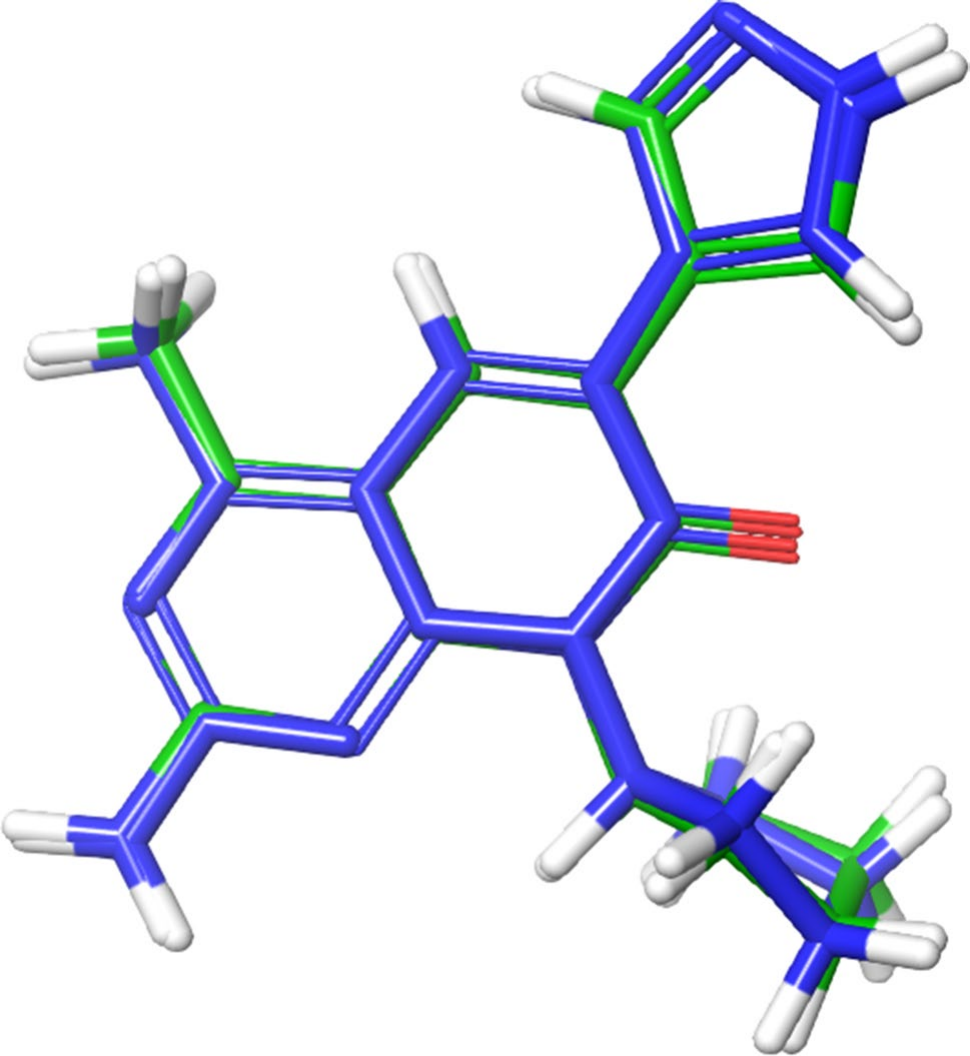
Superimposition of docked (blue) and co-crystallized ligand (green) pose of -2-amino-8-cyclopentyl-4-methyl-6-(1H-pyrazol-4-yl) pyrido[2,3-d] pyrimidin-7(8H)-one of PDB 4FA6 for validating of docking protocol, the observed RMSD was 0.2101

Before screening the ligands using structure-based screening like molecular docking, the docking protocol was validated by redocking the co-crystallized ligand. The RMS deviation was found to be 0.2101, showing that not much change has occurred in the redocked ligand’s pose has, as shown in Fig. 1.

After validation, the screening of 170,408 phytochemicals obtained from the Zinc 15 database was docked using different precision modes described in the methodology. After high-throughput screening (HTVS), 123,940 hits were identified, out of which ligands having docking scores of more than − 7 kcal/mol (5214) were selected for standard precision docking. A further cutoff of − 9 kcal/mol was applied to narrow down the number of ligands to 153, which were then docked by extra precision docking. Among them, the top ten ligands are represented in Table 1. The docking score of the co-crystallized ligand was found to be − 11.076 kcal/mol, and its aminopyrimidine formed hydrogen bonds with the hinge residue Val 88 and Asp 964. Top 10 ligands had docking scores ranging between -14.37 and − 12.67 kcal/mol. Among them, the top ligand was found to be ZINC000014690026 (Salvianolic acid C). So, a literature survey on the compound was done, and it was found that it is a phytoconstituent of a Chinese herb called *Salvia miltiorrhiza,* which also has salvianolic acid A and salvianolic acid B [22]. Both of them halt cancer progression by affecting the cell cycle, causing apoptosis, and adjourning metastasis [23].

**Table 1:** 2D interaction diagrams of inbound ligand, salvianolic acid A, and top ten selected ligands with a summary of docking score, glide energy, and all non-bonding interactions

Sl. no.	ZINC ID	Docking Score (kcal/mol)	MMGBSA dg-bind (kcal/mol)	Interaction diagram	Non-bonding interactions
1	2-amino-8-cyclopentyl-4-methyl-6-(1H-pyrazol-4-yl)pyrido[2,3-d]pyrimidin-7(8H)-one (inbound ligand)	− 11.076	− 56.74		H-bond: Asp 964, Val 882 Charged negative: Glu 880 Charged positive: Lys 890, Lys 833 Polar: Thr 887 Hydrophobic: Met 953, Pro 810, Trp 812, Ala 885, Ile 881, Ile 879, Tyr 867, Phe 961, Ile 963, Ile 831, Met 804
2	ZINC000014690026 (Salvianolic acid C)	− 14.37	− 54.36		H-bond: Asp 964, Lys 890, Asp 841, Tyr 867, Glu 880, Val 882 Salt bridge: Lys 890 Charged negative: Asp 950 Charged positive: Lys 808, Lys 833 Polar: Ser 806, Asn 951 Hydrophobic: Met 953, Pro 810, Trp 812, Ala 885, Ile 881, Ile 879, Leu 838, Phe 961, Ile 963, Phe 865, Ile 831, Leu 969, Met 804, Ala 805
3	ZINC000005004613 (7-glucosyl-luteolin)	− 14.32	− 43.67		H-bonds: Asp 950, Asp 841, Glu 880, Tyr 867, Val 882, Thr 887 Charged positive: Lys 890, Lys 833 Charged negative: Asp 964 Polar: Ser806, Asn 951 Hydrophobic: Met 953, Trp 812, Ala 885, Ile 881, Ile 879, Phe 961, Cys 869, Ile 963, Phe 865, Leu 838
4	ZINC000059728582 (Ambocin)	− 13.92	− 51.02		H-Bond: Asp 950, Lys 807, Asp 964, Glu 880, Val 882 Pi-Pi stacking: Tyr 867 Charged negative: Asp 836 Charged positive: Lys 808, Lys 809, Lys 833 Polar: Ser 806, Asp 951 Hydrophobic: Trp 812, Pro 812, Tyr 757, Met 953, Ala 805, Met 804, Leu 969, Ile 963, Phe 961, Ile 831
5	ZINC000257545754 (Nyasicoside)	− 13.31	− 48.35		H-Bond: Asp 950, Ser 806, Lys 807, Asp964, Lys 833 Pi–Pi cation: Lys 890 Charged negative: Asp 836 Charged positive: Lys 808 Polar: Thr 887, Gln 893, Asn 951 Hydrophobic: Ala 889, Met 953, Met 804, Ala 805, Leu 869, Pro 810, Trp 812, Ile 831, Ile 963, Phe 961
6	ZINC000253532301 (Lonicerin)	− 13.23	− 38.84		H-bond: Lys 833, Asp 964, Lys 890, Val 882, Glu 880, Asp 950, Asn 951, Ser 806 Charged negative: Asp 836 Charged positive: Lys 808, Lys 809 Polar: Gln 893, Thr 897 Hydrophobic: Phe 961, Ile 963, Ile 831, Trp 812, Pro 812, Leu 969, Ala 805, Met 804, Met 953, Ile 879, Ile 881, Tyr 867, Ala 885, Ala 889
7	ZINC000096116102 ((10S)-2-(3,4-dihydroxyphenyl)-5-hydroxy-10-(2-methylpropyl)-9,10-dihydropyrano[2,3-h]chromene-4,8-dione)	− 13.14	− 42.44		H-bond: Lys 890, Val 882, Tyr 867, Asp 841, Asp964 Pi-Pi stacking: Tyr 867 Charged negative: Glu 880 Charged positive: Lys 833 Polar: Thr 867, Ser 806 Hydrophobic: Met 953, Ala 885, Ile 881, Ile 879, Phe 961, Ile 963, Phe 965, Ile 831, Met 804, Pro 810, Trp 812
8	ZINC000095099608 (Tectorigenin 7-O-Xylosylglucoside)	− 13.05	− 39.60		H-Bond: Lys 807, Ser 806, Asp950, Val 882, Glu 880 Pi-Pi stacking: Tyr 867 Charged negative: Asp 836 Charged positive: Lys 808. Lys833 Polar: Gln 893, Asn 951 Hydrophobic: Trp 812, Pro 810, Tyr 757, Ala 805, Leu 969, Ile 963, Phe 961, Ile 831, Ile 879, Ile 881, Met 953
9	ZINC000253532341 (Arillatose B)	− 12.93	− 48.80		H-Bond: Ala 805, Asp 950, Asn 951, Asp 964, Glu 880 Charged positive: Lys 833, Lys 890, Lys 807 Polar: Ser 806, Thr 887 Hydrophobic: Leu 969, Ile 963, Phe 961, Ile 831, Ile 879, Ile 881, Val 882, Ala 885, Trp 812, Pro 810, Tyr 867, Met 804
10	ZINC000096115558 (methyl 2-[2-[(10S)-2-(3,4-dihydroxyphenyl)-5-hydroxy-4,8-dioxo-9,10-dihydropyrano[2,3-h]chromen-10-yl]phenoxy]acetate)	− 12.79	− 43.58		H-Bond: Lys 890, Ser 806, Val 882, Tyr 867, Asp 841, Asp 964 Pi-Pi stacking: Tyr 867 Charged negative: Glu 880, Asp 836, Asp 950 Charged positive: Lys 808, Lys833 Polar: Thr 887, Asn 951 Hydrophobic: Met 804, Pro 810, Trp 812, Leu 969, Ile 831, Met 953, Phe 965, Ile 963, Leu 838, Phe 961, Ile 879, Ile 881, Ala 885
11	ZINC000253500752 (5-[(2R,3S,4R,5R,6S)-6-[[(2R,3S,4S)-3,4-dihydroxy-4-(hydroxymethyl)oxolan-2-yl]oxymethyl]-3,4,5-trihydroxyoxan-2-yl]oxy-4-(3,4-dihydroxyphenyl)-7-methoxychromen-2-one)	− 12.67	− 30.68		H-bond: Lys 890, Asn 951, Asp950, Asp964, Thr 887 Pi-Pi stacking: Tyr 867 Charged negative: Asp 841, Glu 880 Charged positive: Lys 807, Lys 833 Polar: Gln 893, Ser 806 Hydrophobic: Leu 969, Met 953, Ala 889, Ala 805, Met 804, Pro 810, Trp 812, Ile 963, Phe 965, Phe 961, Val 882, Ile 879, Ile 831, Leu 838
12	Salvianolic acid A	− 12.266	− 64.52		H-bond: Asp 964, Ser 806, Glu 880 Pi-Pi stacking: Tyr 867 Charged negative: Asp 950, Asp 836 Charged positive: Lys 808, Lys 833, Lys 802, Lys 890 Polar: Thr 887, Asn 951 Hydrophobic: Met 953, Pro 810, Trp 812, Ile 831, Leu 969, Ile 963, Phe 961, Ile 879, Ile 881, Met 804, Ala 805, Ala 885, Ile 881, Val 882

Both the ligands displayed activity in sensitizing cancer cells to chemotherapy [23]. Therefore, we performed docking and found that Salvianolic acid A and salvianolic acid B have docking scores of − 12.266 and − 6.316 kcal/mol, respectively. MM-GBSA was further used to study the top ten ligands, and salvianolic acid A was selected based on the XP-docking scores representing the binding affinity of the molecules to the 4FA6 PDB protein.

### Ligand-binding energy calculation

The binding energies for the ligand were determined using the Prime-MMGBSA, which indicates the stability of the protein–ligand complex formed after docking. All the top ten ligands showed stability when forming a complex with protein with binding energy greater than − 30 kcal/mol, tabulated in Table 1. The binding energy of the co-crystallized ligand was − 56.74 kcal/mol. The top 10 ligands had scores between − 64.52 and − 30.68 kcal/mol. Salvianolic acid A formed the most stable complex with the protein. Salvianolic acid C had the highest docking score, and less binding energy (− 56.74 kcal/mol) than Salvianolic acid A. This may be due to the ligand energy of salvianolic acid C (0.19 kcal/mol) being more than that of salvianolic acid A (− 13.898 kcal/mol). However, the complex energy for both ligands salvianolic acid C (− 36,388.9 kcal/mol) and salvianolic acid A (− 36,413.1 kcal/mol) was not much different. Further, ADME analysis was performed for the top five ligands and Salvianolic acid A because they have greater than − 30 kcal/mol binding energy.

### ADME analysis

The Qikprop module [24] in Maestro suite predicted the ADME properties of the top ligands by using various descriptors given in Table 2 like QPlogS, QPlogPo/w, QPPCaco, QPlogHERG, % human oral absorption, and Lipinski’s rule of five. All the molecules violated more than one rule of the five, which shows drug-like properties, except for ZINC000014690026 and Salvianolic acid A. The selected ligands had good hydrophilic and hydrophobic balance and good solubility in an aqueous medium, as predicted by QPlogP0/w and QPLogS values, respectively. The QPPCaco and the % human oral absorption were very poor for the molecules. Among all, ZINC000014690026 and Salvianolic acid A had the highest values. The QlogHERG values were more than − 5, except for Salvianolic acid A. Therefore, most ligands have no potential to show inhibition of the HERG potassium channel.

**Table 2:** ADME prediction of the top five selected ligands and salvianolic acid A by using various parameters like solubility, partition, toxicity, absorption, and draggability

Sl. no.	Title	QPlogPo/w	QPlogS	QPlogHERG	QPPCaco	%human oral absorption	Rule of Five
13	ZINC000014690026	2.017	− 4.437	− 4.475	0.709	23.125	1
14	ZINC000005004613	− 1.373	− 2.33	− 4.982	4.007	3.78	2
15	ZINC000059728582	− 1.337	− 2.593	− 6.083	4.699	0	3
16	ZINC000257545754	− 1.337	− 2.244	− 6.055	3.238	2.331	2
17	ZINC000253532301	− 2.155	− 2.739	− 5.878	1.205	0	3
18	Salvianolic acid A	1.379	− 2.938	− 3.452	0.399	14.908	1

### Induced fit docking (IFD)-SP

After ADME analysis, it was proved that the selected molecules had druggable properties. Further, to study how ligand binds to the protein when the protein structure is flexible, induced fit docking studies were performed. IFD helps to overcome the limitations of rigid docking. In IFD, about 20 different poses of each ligand were analyzed. The comparative summary between extra precision docking pose and induced fit docking pose interactions of the ligands has been tabulated in Table 3.

**Table 3:** Summary of the difference between XP docking and induced fit docking pose of the ligands

Sl. No	Drug name	XP-docking pose	IFD pose
19	ZINC000014690026	H-bond: Asp 964, Lys 890, Asp 841, **Tyr 867, Glu 880,** Val 882 Salt bridge: Lys 890 Charged negative: **Asp 950** Charged positive: **Lys 808, Lys 833** Polar: Ser 806, Asn 951 Hydrophobic: Met 953, Pro 810, Trp 812, Ala 885, Ile881, Ile 879, Leu 838, Phe 961, Ile 963, **Phe 865**, Ile 831, Leu 969, Met 804, **Ala 805**	H-bond: Val 882, Lys 890, Asp 964, Asp 841 Salt bridge: Lys 890 Charged negative: **Lys 808, Lys 833** Charged positive: **Glu 880, Asp 950** Polar: Ser 806, Asn 951 Hydrophobic: Leu 838, Ile 963, Phe 961, Phe 965, Leu 969, Met 804, Tyr 867, Ile 831, Ile 879, Ile 881, Ala 885, Trp 812, Pro 810, Met 953
20	ZINC000005004613	H-bonds: Asp 950, Asp 841, **Glu 880,** Tyr 867, Val 882, **Thr 887** Charged positive: **Lys 890, Lys 833** Charged negative: **Asp 964** Polar: Ser806, Asn 951 Hydrophobic: Met 953, Trp 812, Ala 885, Ile 881, Ile 879, Phe 961, **Cys 869,** Ile 963, Phe 865, Leu 838	H-bond: Val 882, Asp 964, Asp 841, Tyr 867, Asp 950 Charged negative: **Lys 833** Charged positive: **Glu 880** Polar: Ser 806, Asn 951, **Thr 887** Hydrophobic: Leu 838, Ile 963, Phe 961, **Phe 965**, **Leu 969**, **Met 804**, **Tyr 867, Ile 831,** Ile 879, Ile 881, Ala 885, Trp 812, **Pro 810**, Met 953
21	ZINC000059728582	H-Bond: Asp 950**, Lys 807**, Asp 964, **Glu 880**, Val 882 Pi-Pi stacking: Tyr 867 Charged negative: **Asp 836** Charged positive: **Lys 808, Lys 809, Lys 833** Polar: **Ser 806,** Asp 951 Hydrophobic: Trp 812, Pro 812, Tyr 757, Met 953, Ala805, Met 804, Leu 969, Ile 963, Phe 961, Ile 831	H-bond: Val 882, Asp 964, Asp 841, Tyr 867, Asp 950 Pi-cation: **Lys 833** Pi-Pi stacking: Tyr 867 Charged negative: **Lys 808, Lys 807** Charged positive: **Asp 964, Asp 836** Polar: Asn 951 Hydrophobic: Leu 969, Met 804, Ile 831, Pro 810, Trp 812, Ile 879, Ile 881, Val 882, Phe 961, Ile 963, Met 953
22	ZINC000257545754	H-Bond: **Asp 950**, Ser806, **Lys 807**, Asp964, **Lys 833** Charged negative: **Asp 836** Charged positive: **Lys 808** Polar: **Thr 887, Gln 893,** Asn 951 Hydrophobic: **Ala 889,** Met 953, Met 804, Ala 805, Leu 869, Pro 810, Trp 812, Ile 831, Ile 963, Phe 961	H-bond: **Val 882**, **Ala 885**, Asp 964, **Asp 841, Tyr 867, Lys 808**, Ser 806, Asp964 Pi-Pi stacking: **Trp 812** Charged negative: **Lys 833, Lys 890** Charged positive: **Asp 950, Glu 880** Polar: Asn 951 Hydrophobic: Met 953, **Ile 881, Ile 879, Ile 831,** Pro 810, **Leu 838, Cys 869, Leu 969, Phe 965,** Ile 963
23	ZINC000253532301	H-bond: **Lys 833**, Asp 964, **Lys 890**, **Val 882**, **Glu 880**, Asp 950, Asn 951, **Ser 806** Charged negative: **Asp 836** Charged positive: **Lys 808, Lys 809** Polar: **Gln 893, Thr 897** Hydrophobic: **Phe 961**, Ile 963, Ile 831, Trp 812, **Pro 812**, Leu 969, **Ala 805,** Met 804, Met 953, Ile 879, **Ile 881, Tyr 867, Ala 885,** Ala 889	H-bond: **Asp 841**, **Tyr 867,** Asp 964, Asn 951, Asp 950**, Lys 808** Charged negative: **Lys 833, Lys 890, Lys 807** Polar: **Thr 887, Ser 806** Hydrophobic: Met 953, Ala 889, Trp 812, Ile 879, Ile 831, **Phe 965**, Ile 963, Leu 969, Met 804
24	Salvianolic acid A	H-bond: Asp 964, **Ser 806, Glu 880** Pi-Pi stacking: **Tyr 867** Charged negative: **Asp 950,** Asp 836 Charged positive: **Lys 808, Lys 833, Lys 802, Lys 890** Polar: Thr 887, **Asn 951** Hydrophobic: Met 953, Pro 810, Trp 812, Ile 831, **Leu 969**, Ile 963, Phe 961, Ile 879, Ile 881, Met 804, **Ala 805, Ala 885, Ile 881, Val 882**	H-bond: Asp 964, **Asp 841, Tyr 867, Lys 833, Lys 890, Ala 885, Val 882** Pi-Pi stacking: **Tyr 812** Charged negative: Asp 836, **Glu 880** Polar: **Thr 886**, Thr 887 Hydrophobic: Met 804, Pro 810, Ile 831, **Phe 965, Leu 838**, Ile 963, Phe 961, **Cys 869**, Ile 879, Ile 881, Met 953

In the case of ZINC000014690026 (Salvianolic acid C), H-bonds with Tyr 867 and Glu 880 were observed in the XP-docking pose, which was absent in the IFD. ZINC000005004613 had an additional H-bond with Glu 880 and Thr 887 during glide XP docking, which is not seen in IFD. For Ambocin, additional H-bond with Glu 880 and Lys 807 were observed, which were not present in the IFD pose, but were seen in XP-docking pose; there is a Pi–cation interaction with Lys 833, which is an essential amino acid for inhibitory activity of the ligand. The compound ZINC000257545754 showed new H-bonds with Val 882, Ala 885, Asp 841, Tyr 867, Lys 808, and a Pi-Pi stacking interaction with Trp 812 in IFD pose. In the case of Salvianolic acid A, additional H-bonds with Asp 841, Tyr 867, Lys 833, Lys 890, Ala 885, Val 882, and Pi-Pi stacking interaction with Tyr 812 were observed in the IFD pose, which indicates strong interactions with the binding site.

**Fig. 2:**
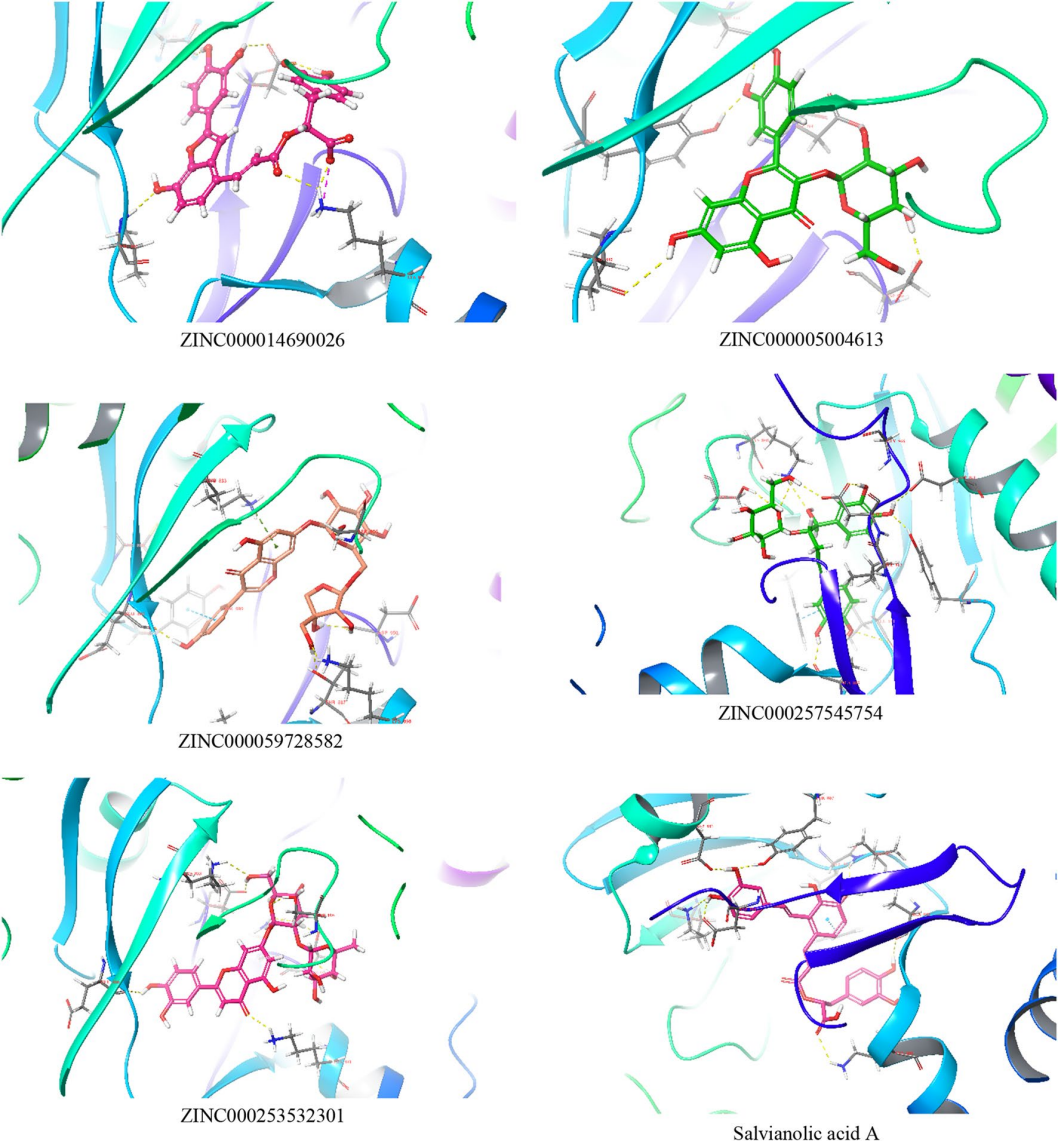
3D poses of ligands with the protein used for MD simulation showing the hydrogen bond (yellow dotted line), Pi–Pi stacking (blue dotted line), Pi–cation (green dotted line), and salt bridge (lavender dotted line)

For further MD studies, IFD poses in which the ligand showed maximum interactions with essential amino acids (Lys 833, Val 882) in the binding site were selected for 100 ns MD simulation, as shown in Fig. 2.

### WaterMap analysis for hydration site prediction

Water map analysis involves the calculating enthalpy and entropy of every hydration site relative to bulk solvent by using thermodynamic molecular dynamics simulation [25]. WaterMap analysis considers the theory of Lazaridis and Karplus, that considers a significant contribution of solvent reorganization energy and entropy to the solvation-free energy in an inhomogeneous system. WaterMap lets us determine the presence of a hydration site with an easily replaceable high-energy hydration site, represented in red spheres. The ones with less energy are represented as green spheres tightly placed around the ligand. A holistic approach was used to determine the relation between the binding affinity of the hit molecules and the computed hydration site energetics quantitatively.

Analyzing the apoprotein area (around 5Ǻ of the ligand-binding pocket) revealed the presence of 34 water molecules. The drug docking pocket contains water molecules with the hydration energy ranging from − 4.5 to 4.97 kcal/mol with the occupancy ranging from 1 to 0.28, indicating the wide range of water molecules around the pocket, as shown in Table 4.

**Table 4:** Predicted hydration site, occupancy, and thermodynamic properties of the available hydration sites within five angstroms selected ligands

Site	Occupancy	ΔH (kcal mol-1)	− T Δ S (kcal mol-1)	ΔG (kcal mol-1)
4	1	− 9.64	5.14	− 4.5
8	0.98	− 4.83	4.22	− 0.61
13	0.92	− 4.82	3.26	− 1.56
20	0.87	− 1.69	3.37	1.68
24	0.83	− 3.7	2.87	− 0.83
25	0.82	− 2.77	3.02	0.25
26	0.82	− 0.12	2.74	2.62
32	0.78	2.5	2.79	5.29
37	0.7	2.58	2.13	4.71
39	0.69	− 5.39	2.38	− 3.01
43	0.66	− 0.38	2.02	1.64
49	0.61	1.98	2.15	4.13
52	0.61	− 2.3	1.97	− 0.33
59	0.54	− 5.61	2.02	− 3.59
60	0.53	− 1.24	1.65	0.41
64	0.5	2.06	1.55	3.61
67	0.49	− 0.53	1.4	0.87
69	0.49	− 2.91	1.49	− 1.42
70	0.48	− 0.42	1.46	1.04
73	0.46	2.21	1.33	3.54
75	0.46	− 1.1	1.38	0.28
77	0.45	3.5	1.34	4.84
81	0.44	− 0.89	1.35	0.46
83	0.42	0.3	1.17	1.47
85	0.4	0.48	1.23	1.71
97	0.34	4.02	0.95	4.97
98	0.34	0.22	0.92	1.14
102	0.32	− 0.55	0.87	0.32
104	0.31	− 4.88	1	− 3.88
105	0.3	− 0.96	0.85	− 0.11
106	0.29	1.17	0.82	1.99
107	0.29	0.2	0.85	1.05
110	0.29	− 0.58	0.79	0.21
116	0.28	0.06	0.78	0.84

All the ligands were found to cover the hydrophobic pockets formed by the hydration sites 37, 32, 73, 77, 72, and 64. The hydrophilic pocket of the protein comprises hydration sites 4,13,59,104, 60, which have less negative energy indicating strong binding. The predicted overlaps greater than 0.4, and the available hydration sites within 5 angstroms of the selected top ligand in the docking site of 4FA6 have been tabulated in Table 5. Compound ZINC000014690026 contains 17 water molecules which are in contact or proximity with ligand to that of relative free energy between 4.84 and − 3.59 kcal/mol. The benzyl group of ligands makes π–stacking interaction, surrounded by the hydration sites 32, 37, 73, and 77 with positive binding free energy. These hydration sites 32, 37, 73, and 77 have positive free energy indicating the possibility of easy replacement of these hydration sites by the ligand.

**Table 5:** Predicted hydration site and overlaps for the selected top ligand in the docking site of 4FA6

Sl no.	Name	Site and overlap	Diagram
25	ZINC000014690026	8(1), 13(0.86), 20(1), 24(0.43), 26(1), 32(1), 37(1), 43(1), 59(0.47), 67(0.89), 73(1), 77(1), 81(0.65), 83(1), 98(1), 106(1), 116(1)	
26	ZINC000005004613	8(1), 20(1), 24(1), 25(0.83), 32(1), 37(1), 43(1), 59(0.52), 73(1), 77(1), 81(1), 98(0.51), 105(1), 110(0.5)	
27	ZINC000059728582	24(1), 26(1), 32(1), 37(1), 43(1), 52(0.73), 59(0.56), 60(1), 69(1), 70(1), 73(1), 75(1), 77(1), 85(0.6), 97(1), 98(0.92), 102(0.72), 104(1), 106(1), 110(0.55)	
28	ZINC000257545754	4(1), 13(0.63), 25(0.74), 26(0.96), 32(1), 37(1), 39(1), 59(1), 60(0.56), 70(0.55), 73(1), 75(0.66), 77(1), 81(0.79), 85(0.66), 98(0.51), 104(1), 105(1), 106(0.45), 107(1), 116(0.43)	
29	ZINC000253532301	4(1), 13(0.7), 26(1), 32(1), 37(1), 39(0.8), 52(0.69), 59(1), 60(0.61), 64(0.59), 69(1), 73(1), 75(0.55), 77(1), 83(1), 85(0.45), 98(1), 102(1),104(0.84), 105(0.62), 106(0.96), 107(0.87), 110(1), 116(1)	
30	Salvianolic acid A	4(0.57), 24(1), 25(0.77), 26(1), 32(1), 37(1), 43(1), 59(1), 67(0.66), 70(0.68), 73(1), 77(1), 81(0.81), 83(1), 102(0.47), 104(0.63), 110(1), 116(1)	

Similarly, 14 water molecules are present around the ligand ZINC000005004613 with occupancy in the range of 0.3–0.98 kcal/mol and ΔG with free energy from 5.29 to − 3.59 kcal/mol. Most hydration sites around this ligand have positive free energy except for sites 8, 24, and 59 (− 3.59 kcal/mol). Compound ZINC000059728582 contains 20 hydration sites with free energy ranging from 4.97 to − 3.88 kcal/mol. Compound ZINC000257545754 has 21 hydration sites with free energy ranging from 4.84 to − 4.5 kcal/mol, compound ZINC000253532301 contains 24 hydration sites with free energy ranging from 5.29 to − 4.5 kcal/mol, and Salvianolic acid A has 18 hydration sites with free energy ranging from 5.29 to − 4.5 kcal/mol.

### Molecular dynamics simulation

**Fig. 3:**
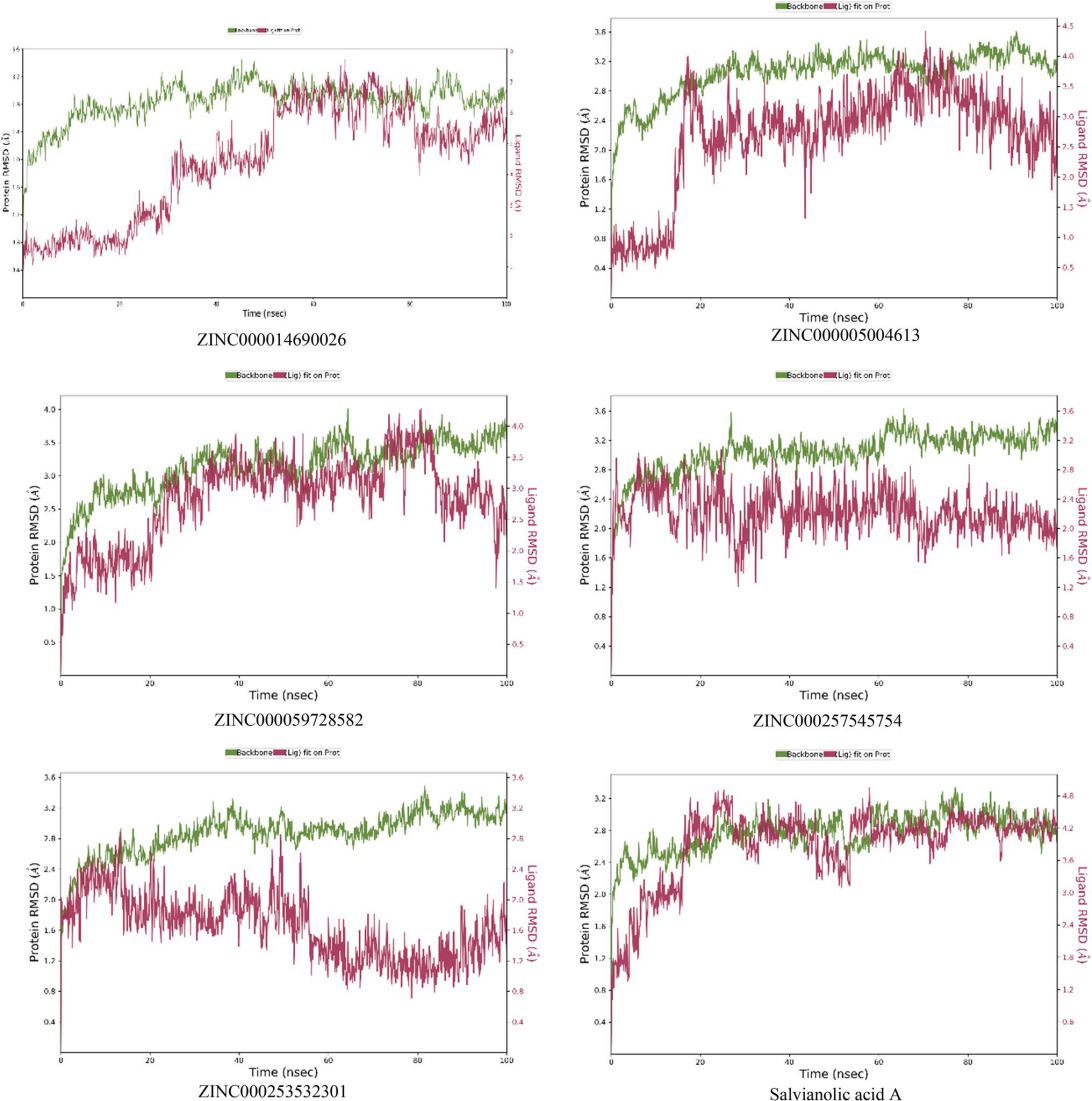
RMSD plots of protein and ligand after MD simulation for 100 ns

**Fig. 4:**
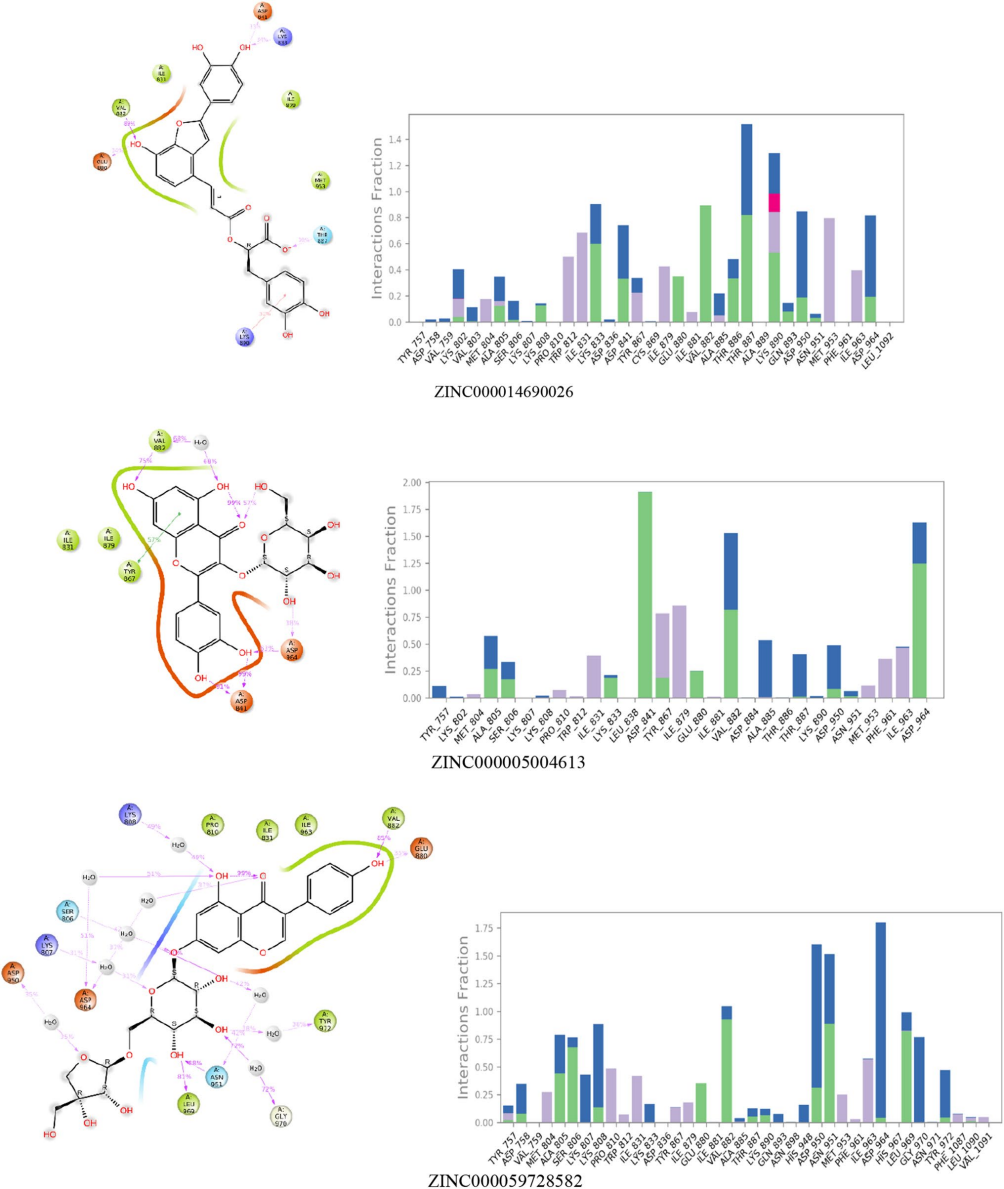

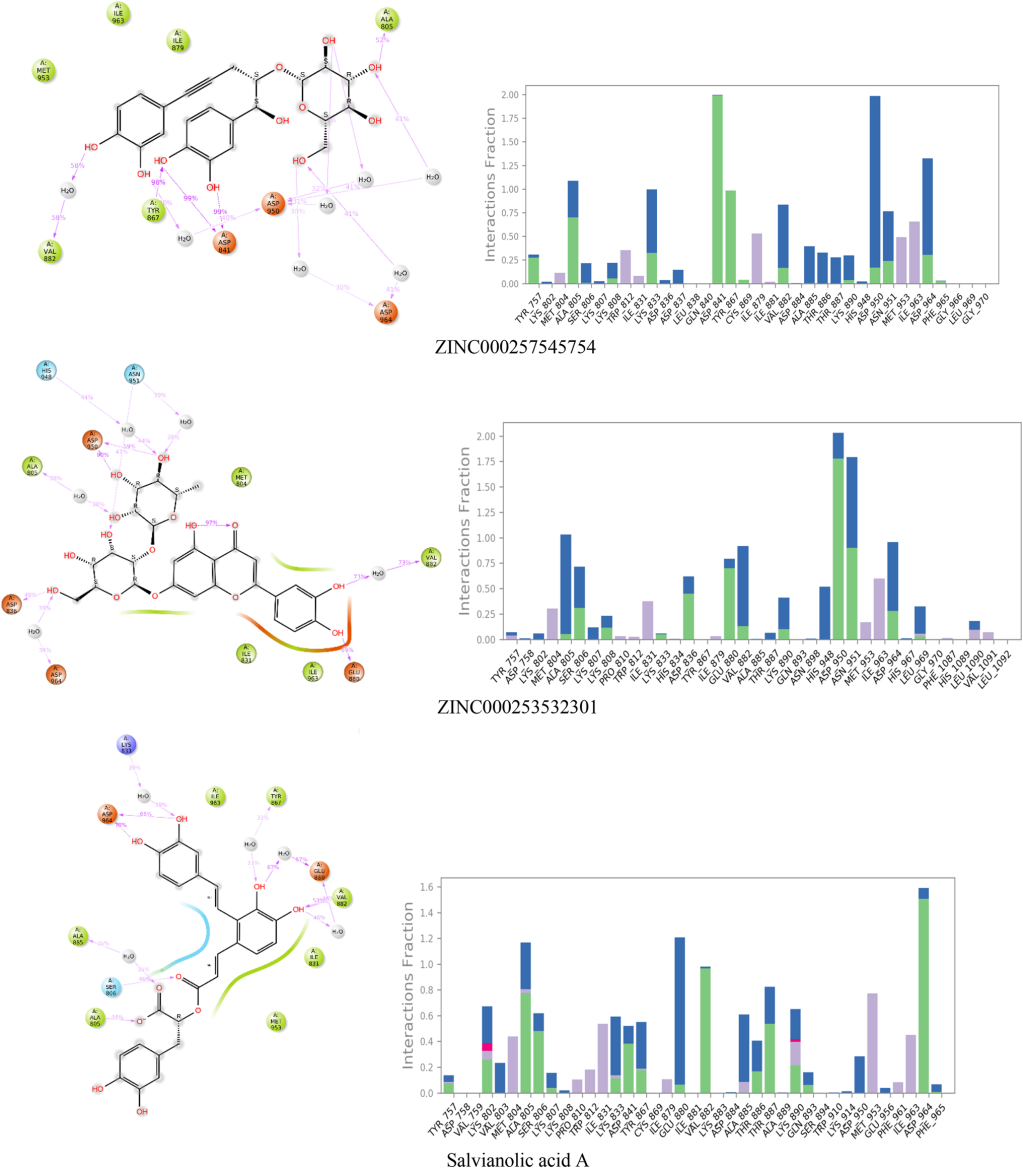
2D interaction diagram with protein–ligand contacts for 100 ns MD simulation in which green represents H-bonds, pink represents an Ionic bond, blue represents a water bridge, and gray represents hydrophobic interaction

The deviation of the complex from the reference frame which is indicated by root mean square deviation has been shown for the top five ligands with Salvianolic acid A in Fig. 3. 2D interaction diagram of the ligand after MD simulation, showing the percentage of time the amino acid had interacted with the ligand, fraction of interaction, and type of interaction, is shown in Fig. 4.

The protein–ligand ZINC000014690026 complex system contains 100,299 atoms, which includes 28,567 water molecules and eight sodium molecules to neutralize the charge of the system. The RMSD plot of protein was stable throughout the simulation, with RMSD varying between 2 and 3.6 Ǻ. But, a wide deviation in the ligand was seen till 50 ns, after which it was stabilized with RMSD ranging from 4 to 8 Ǻ. The ligand stabilization after 50 ns was due to the formation of an additional bond with Lys 802. The critical residue Val 882 showed hydrogen bond interaction for 89% duration with the hydroxyl group of ZINC000014690026. Interactions with the residues Asp 841, Glu 880, Val 882, and Lys 890 observed in XP docking were retained during MD simulation, but interactions like Asp 964 and Tyr 867 were not seen. Instead, interaction with Thr 887 and Lys 833 was observed.

The protein–ligand ZINC000005004613 complex system contains 100,290 atoms, of which the water molecules were 28,565. The system neutralization was done with seven sodium ions. RMSD plot showed stability after 20 ns for the entire simulation. The drift in the RMSD might be due to the disappearance of H-bond and water bridge interactions with residue Ser 806, Asp 950, hydrophobic interaction with residues Ile 831, Met 95. New interactions with residue Lys833, Glu 880, and Phe 961 were also formed after 20 ns. The important residue Val 882 showed hydrogen bond interaction for 75% duration and water bridge interaction for 68% duration with the hydroxyl group of ZINC000005004613. The interactions with residues Asp 950, Glu 880, and Thr 887, which were observed in the XP docking, were lost during MD simulation.

Ligand ZINC000059728582-protein complex consisted of 100,290 atoms, including 28,560 water molecules and seven sodium atoms to neutralize the charge. RMSD plot showed stable ligand RMSD in the range 1.5–2 Ǻ till 20 ns, followed by a steep increase ranging from 2.5 to 3.5 Ǻ till 80 ns, then a fall in the range of 2–3 Ǻ. The protein showed stable RMSD for the entire duration with the range of 2–3.5 Ǻ. The change in the RMSD after 20 ns might be due to the hydrophobic interactions’ loss with the residues Tyr 867, Ile 879, and hydrogen bond interaction with Glu 880. Formation of the new interactions was observed with the residues Asn 951, Leu 969, Ser 806, Lys 808, Gly 970, and Tyr 972. The hydroxyl group of the ligand formed a hydrogen bond with the key residue like Val 882 for 85% of the simulation time.

Ligand ZINC000257545754-protein complex consisted of 100,312 atoms, including 28,570 water molecules and seven sodium atoms for neutralization of the charge. RMSD plot showed a stable ligand RMSD for the entire 100 ns with RMSD ranging from 1.2 to 2.8 Ǻ. Similarly, protein also had stable RMSD for the simulation period, showing RMSD ranging from 2.4 to 3.2 Ǻ. A stable water bridge and hydrogen bond interaction was formed for 58% of the simulation with the key residue Val 882. Loss of interactions with residues Ser806, Lys807, Lys 833, and Glu880 was observed in XP docking, and the formation of new interactions with residues Val 882, Tyr 867, Asp841, and Ala805 was seen during the simulation.

Ligand ZINC000253532301-protein complex consisted of 100,360 atoms, including 28,582 water molecules and six sodium atoms for neutralization. RMSD plot for protein shows the stability with the deviation in the range 2.8–3.6 Ǻ, and the ligand showed a deviation in the range 1–2.8 Ǻ. After 15 ns, the ligand showed a fall in RMSD from 2.8 Ǻ, which might be due to the loss of interaction with Tyr 757, Asp 836, His 948, and Asn 964 residues. However, after 20 ns duration, a stable RMSD plot was seen with the ligand. Key residue Val 882 formed water-linked hydrogen-bonding interaction for 73% duration of the simulation. Formation of new hydrogen-bonding interactions with residues His948, Ala 805, and Asp836 and loss of the interactions with residues Lys 833, Lys 890, and Ser 806 were observed in extra precision docking during MD simulation.

The protein–ligand Salvianolic acid A complex used for the simulation contained 28,560 water molecules, and eight sodium ions to neutralize the system. The protein was found stable during the entire simulation period, with RMSD ranging from 2 to 2.8 Ǻ. The complex was stable after 20 ns for the whole period of MD simulation with ligand RMSD ranging from 3.6 to 4.2 Ǻ. Change in the RMSD after 20 ns might have occurred due to the loss of hydrophobic and salt bridge interaction with residues Asp 841, Thr 887, and Ile879 and formation of strong interactions with residues Lys 802, Glu 880, and Asp 964. All the interactions seen in extra precision docking were seen in MD simulation with other new interactions with residues Ala 805, Ala 885, Lys 833, Asp 964, Tyr 867, and Val 882.

### Thermal MM-GBSA

**Fig. 5:**
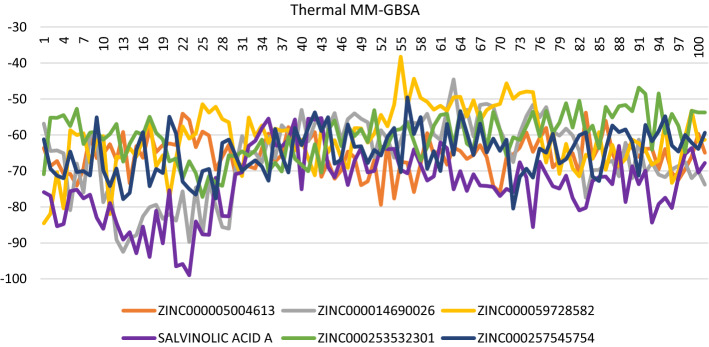
Ligand-binding energy of top five ligands and salvianolic acid A obtained from every 10th frame of 1002 frames after running 100 ns MD simulation

The trajectory generated after MD simulation was used for Thermal MM-GSBA. The average binding energy was more than − 60 kcal/mol for all the ligands. The most stable complex among all was Salvianolic acid A with − 73.87 ± 9.56 kcal/mol, which also showed a stable trajectory during MD simulation. The stability was due to interactions with Ala 805, Ala 885, Lys 833, Asp 964, Tyr 867, and Val 882. The binding energies of the top 5 ligands and salvianolic acid A are tabulated in Table 6, and variation in the binding energy of structure obtained from the various frames of MD simulation is shown in Fig. 5.

**Table 6:** Binding energy of molecules post MD simulation; the value represented here is the average of energy calculated from every 10th frame of 1000 frames obtained from MD simulation

Sl. no.	Name	MMGBSA dG bind	Ligand energy	Complex energy	Receptor energy
31	ZINC000014690026	− 66.08 ± 10.68	− 3.33 ± 2.82	− 27,567.7 ± 123.71	− 27,498.3 ± 122.33
32	ZINC000005004613	− 65.67 ± 4.99	− 192.11 ± 2.44	− 27,792.7 ± 111.23	− 27,535 ± 110.92
33	ZINC000059728582	− 60.74 ± 8.48	− 185.51 ± 2.68	− 27,684.3 ± 105.85	− 27,438.1 ± 107.02
34	ZINC000257545754	− 64.82 ± 6.36	41.65 ± 2.71	− 27,511.8 ± 131.33	− 27,488.6 ± 132.79
35	ZINC000253532301	− 60.42 ± 6.30	− 181.01 ± 1.64	− 27,662.6 ± 110.30	− 27,421.1 ± 109.33
36	Salvianolic acid A	− 73.87 ± 9.56	− 8.62 ± 3.19	− 27,642.1 ± 119.78	− 27,559.6 ± 119.39

## Conclusion

Targeting PI3K and mTOR can synchronously inhibit both upstream and downstream signaling proteins in the pathway. A single PI3K/mTOR dual inhibitor has an advantage of causing less drug resistance. Therefore, in this study, in silico studies were opted for by using stratified approaches like HTVS, SP, and XP docking of phytoconstituents obtained from curated databases like Zinc 15. After the docking, MM-GBSA, ADME prediction, WaterMap, and molecular dynamics prediction were performed. By molecular docking, it was found that Salvianolic acid C had the highest docking score, and therefore, we carried out a literature survey and found that Salvianolic acid A has a similar structure to it and it was already proven to suppress tumor growth in acute myeloid leukemia by inhibiting PI3K/AKT pathway. However, the molecular mechanism by which it acts has not been established. Therefore, molecular dynamics was performed to predict if the top five ligands and salvianolic acid A form a stable complex with the PI3K protein or not. In 100 ns MD, ZINC000059728582, ZINC000257545754, ZINC000253532301, and Salvianolic acid A showed very stable complex, which can be seen in their RMSD values throughout the simulation. ZINC000014690026 showed interaction with Val 882 for more than 89% of the time, proving that it occupies the same pocket as that of the known inbound ligand of 4FA6. From the current study, it could be concluded that Salvianolic acid A can be explored for molecular mechanism by which it showed tumor suppression effect in acute myeloid leukemia. Other ligands mentioned can further be evaluated by various in vitro and in vivo studies in various types of cancer which involves PI3K/mTOR pathway.
